# Supplementary material for: Cell-specific and divergent roles of the CD40L-CD40 axis in atherosclerotic vascular disease
Source: Nat Commun. 2021 Jun 18;12:3754. doi: 10.1038/s41467-021-23909-z (PMC8213756; doi:10.1038/s41467-021-23909-z)
Supplement: Supplementary file 1 — Supplementary Information [file 41467_2021_23909_MOESM1_ESM.pdf]

Supplementary Information

Cell-specific and divergent roles of the CD40L-CD40 axis in atherosclerotic vascular disease

Lacy, Bürger, Shami et al.

This PDF file includes:
Supplementary Figures
Supplementary Tables

Supplementary Figures

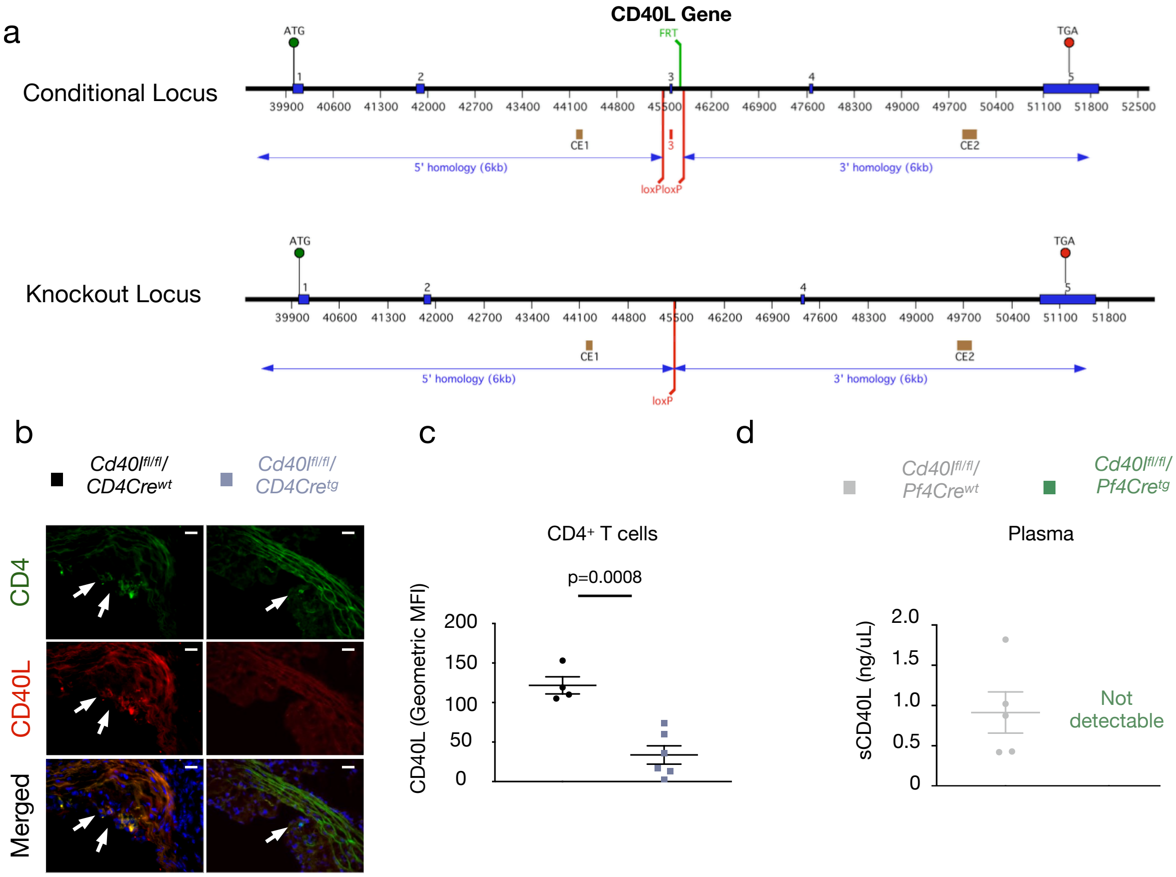

Supplementary Figure 1 Conditional CD40L knockout model generation and confirmation. **a** Model for generating CD40L conditional knockout mice displaying exons in blue rectangles along the genome. Initiation (ATG) and termination (TGA) are depicted as green and red lollipops, respectively. Exon 3 is flanked by loxP sites in the conditional locus, which can be excised by cre recombinases as displayed in the knockout locus. Two conserved intronic sequences named CD1 and CD2 (brown rectangles) remain untouched. **b-d**, Confirmation of knockout models with **b** immunohistochemistry of aortic root plaques staining CD40L (red) expression on CD4+ T cells (green) along with nuclear DAPI staining (blue) in the *Cd40l*$^{fl/fl}$/*Cd4Cre* model (scale bar: 25 µm), **c** flow cytometric analysis of the CD40L expression on activated CD4+ T cells in the *Cd40l*$^{fl/fl}$/*Cd4Cre* model [n: WT=4, TG=6], and **d** protein quantification of soluble CD40L (sCD40L) in the plasma from *Cd40l*$^{fl/fl}$/*Pf4Cre*^{tg} and their wild type littermates [n=5]. Data are represented as mean ± s.e.m and n refers to biologically independent animals. Data was analyzed by a two-tailed unpaired Student's *t* test (**c-d**). Source data are provided as a Source Data file.

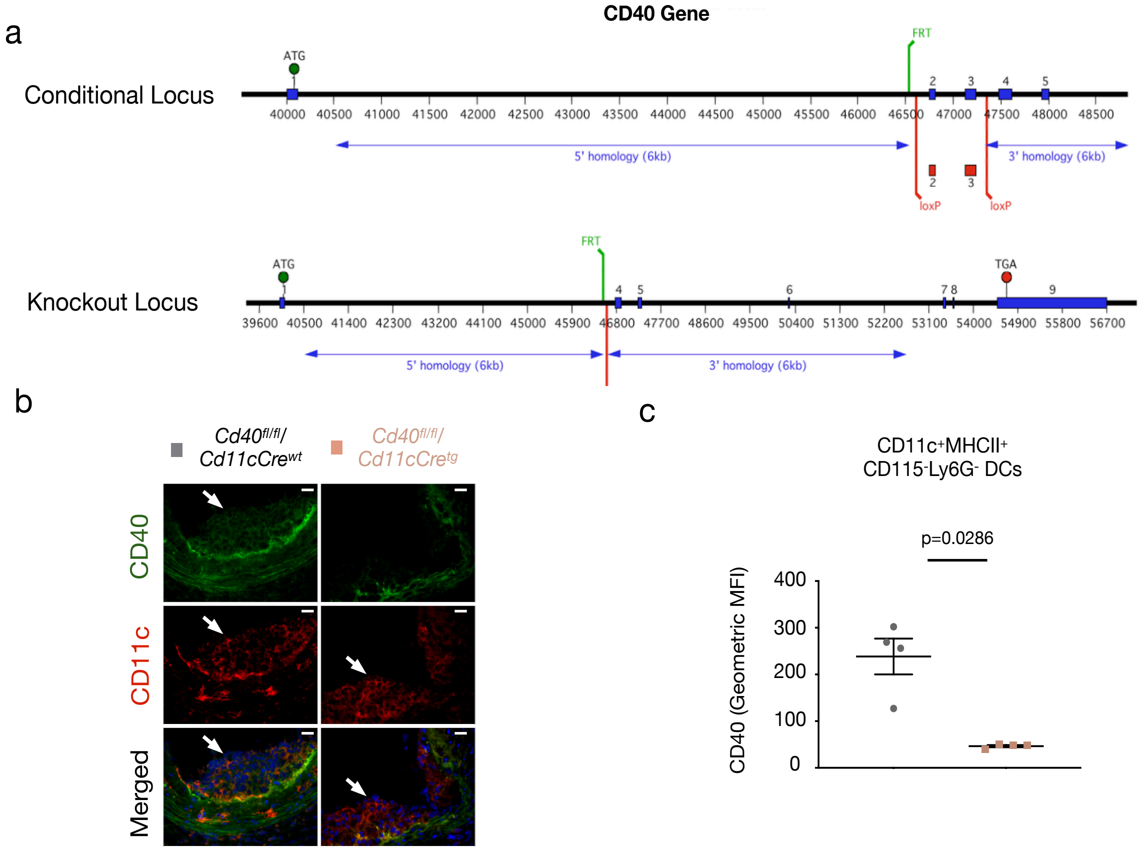

Supplementary Figure 2 Conditional CD40 knockout model generation and confirmation. **a** Model for generating CD40 conditional knockout mice displaying exons in blue rectangles along the genome. Initiation (ATG) and termination (TGA) are depicted as green and red lollipops, respectively. Exons 2 and 3 are flanked by loxP sites in the conditional locus, which can be excised by cre recombinases as displayed in the knockout locus. **b-c**, Confirmation of knockout models with **b** immunohistochemistry of aortic root plaques staining CD40 (green) expression on CD11c+ DCs (red) along with nuclear DAPI staining (blue) in the *Cd40fl/fl/Cd11cCre* model (scale bar: 25 µm) and **c** flow cytometric analysis comparing the CD40 expression on splenic dendritic cells isolated from *Cd40fl/fl/Cd11cCretg* and their wild type littermates [n=4]. Data are represented as mean ± s.e.m and n refers to biologically independent animals. Data was analyzed by a two-tailed Mann Whitney test (**c**). Source data are provided as a Source Data file.

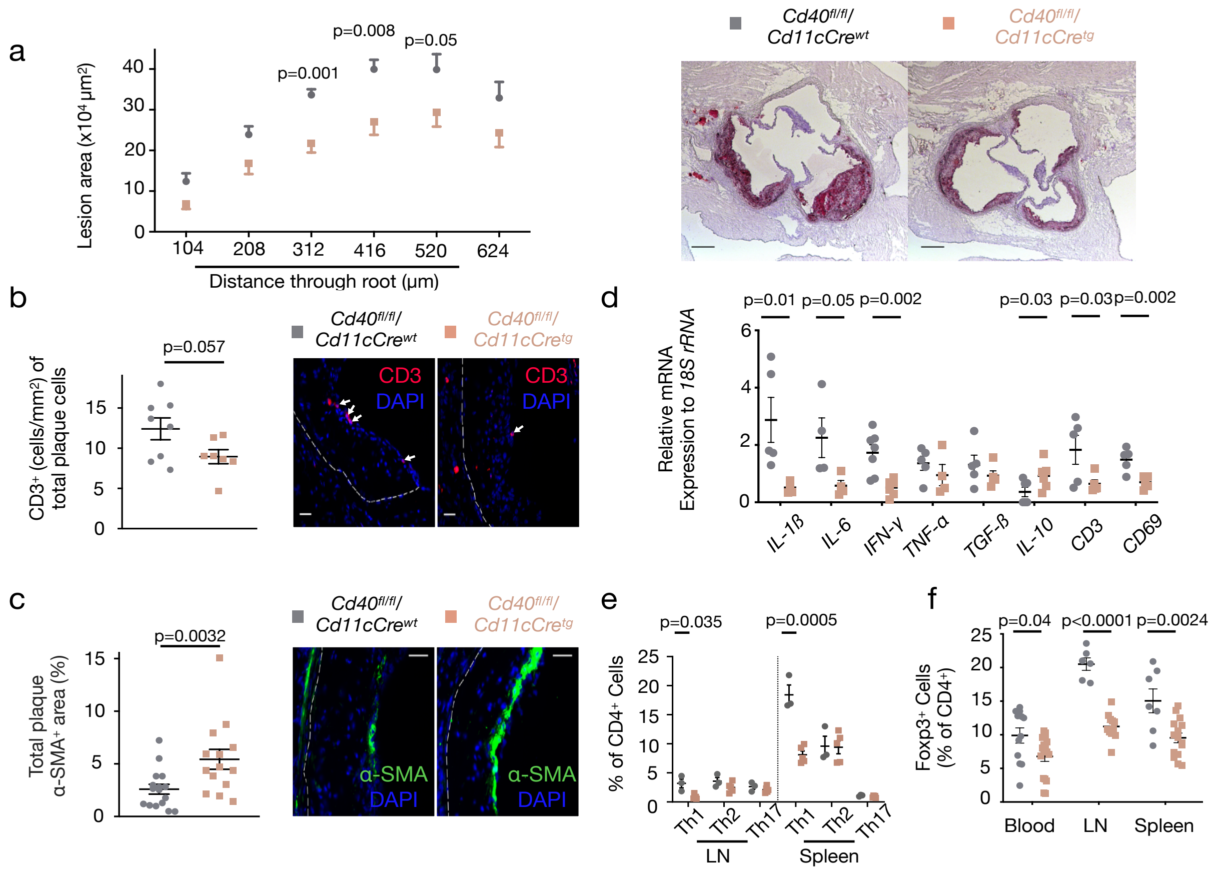

Supplementary Figure 3 DC-specific CD40 knockout model phenocopies T cell specific CD40L model. **a-c**, Plaque phenotyping using Oil Red O and immunofluorescent staining from the aortic root of *$Cd40^{fl/fl}$/Cd11cCre* mice. **a** Atherosclerotic plaque area in cross-sections at indicated positions of the aortic root from *$Cd40^{fl/fl}$/Cd11cCre* mice [n: WT=8, TG=12] together with representative photomicrographs showing Oil Red O-stained sections (scale bar: 200 µm). **b** Analysis of $CD3^+$ T cells (red) as well as nuclear DAPI (blue) staining (right) together with representative images (left, scale bar: 25 µm) [n: WT=8, TG=7]. **c** Analysis of α-SMA^+ content (green) as well as nuclear DAPI (blue) staining (right) together with representative images (left, scale bar: 25 µm) [n: WT=16, TG=14]. **d** Gene expression analysis using qPCR to evaluate various mRNA cytokine expression profiles in the descending aorta of *$Cd40^{fl/fl}$/Cd11cCre* mice [n=4-7]. **e** Flow cytometric analysis of effector T cell populations including Th1 ($CD62L^-CD44^+CXCR3^+CCR6^-$), Th2 ($CD62L^-CD44^+CXCR3^-CCR6^-$), and Th17 ($CD62L^-CD44^+CXCR3^-CCR6^+$) in the LNs [n: WT=3, TG=5] and spleen [n: WT=3, TG=5]. **f** Flow cytometric analysis of Treg ($CD4^+Foxp3^+$) populations in the blood [n: WT=12, TG=17], lymph nodes [n: WT=6, TG=9], and spleen [n: WT=7, TG=15]. Data are represented as mean ± s.e.m and n refers to biologically independent animals. Data was analyzed by either a two-tailed unpaired Student's *t* test (**a-b, d-f**) or Mann Whitney test (**c-f**). Interleukin-1 beta, IL-1ß; Interleukin-6, IL-6; Interferon-gamma, IFN-γ; Tumor necrosis factor-alpha, TNF-α; Transforming growth factor-beta, TGB-ß; Interleukin-10, IL-10; Cluster of differentiation 3, CD3; Cluster of differentiation 69, CD69.Source data are provided as a Source Data file.

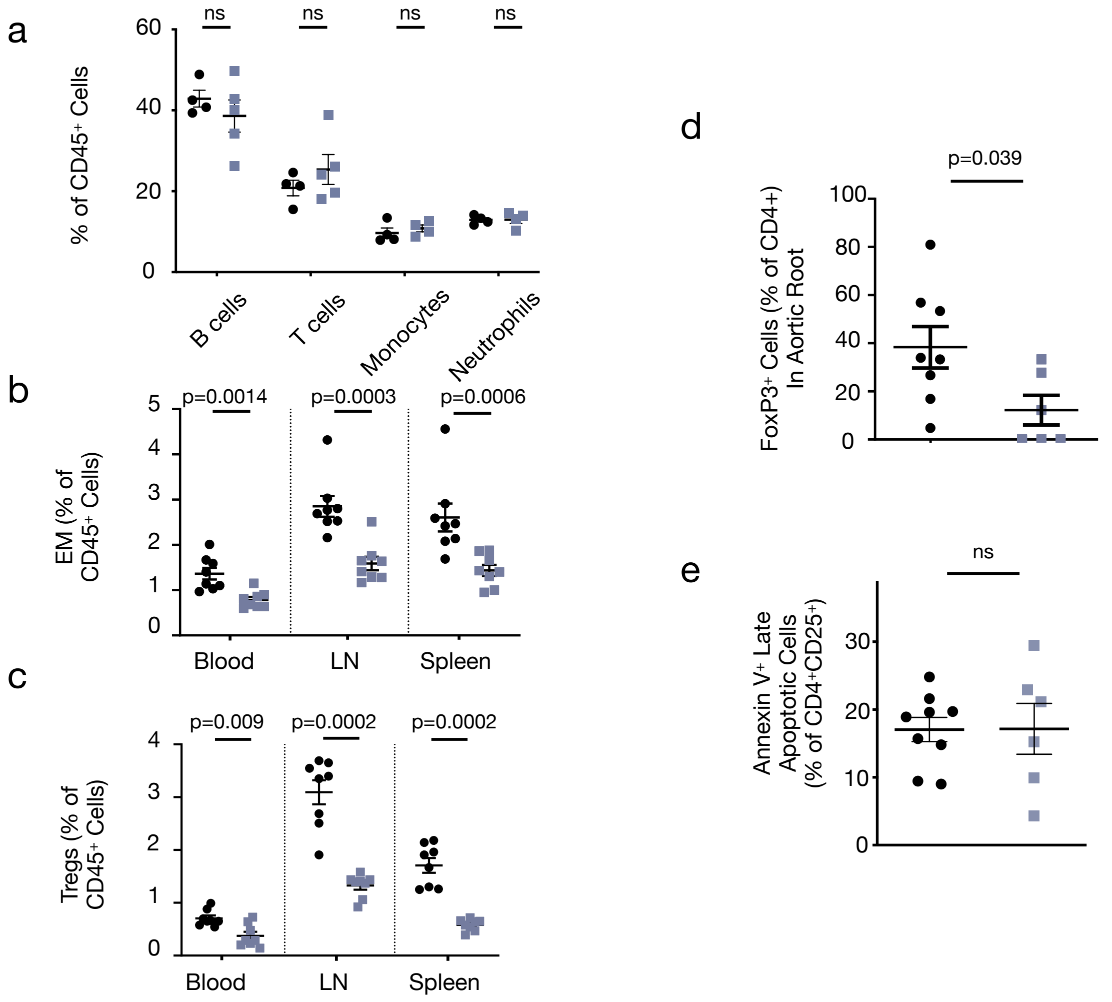

Supplementary Figure 4 T cell CD40L deficiency alters Treg and Effector Memory populations. **a** Flow cytometric analysis of major immune cell populations in the blood of *Cd40l fl/fl/Cd4Cre^{tg}* and their wild-type littermates [n: WT=4, TG=5]. **b-c,** Flow cytometric analysis of **b** Effector Memory (EM) and (**c**) T regulatory (Tregs) cells as a function of all white blood cells ($CD45^+$) [n=8]. **d** Analysis of immunohistochemistry staining of the percentage of $Foxp3^+$ populations within all $CD4^+$ T cells in the aortic root [n: WT=8, TG=6]. **e** Flow cytometric analysis of apoptotic, Annexin-V^+ Tregs ($CD4^+CD25^+$) isolated from the thymus in *Cd40l$^{fl/fl}$/CD4cre* mice [n: WT=9, TG=6]. Data are represented as mean ± s.e.m and n refers to biologically independent animals. Data was analyzed by either a two-tailed unpaired Student's *t* test (**a-b, d-e**) or Mann Whitney test (**b-c**). Source data are provided as a Source Data file.

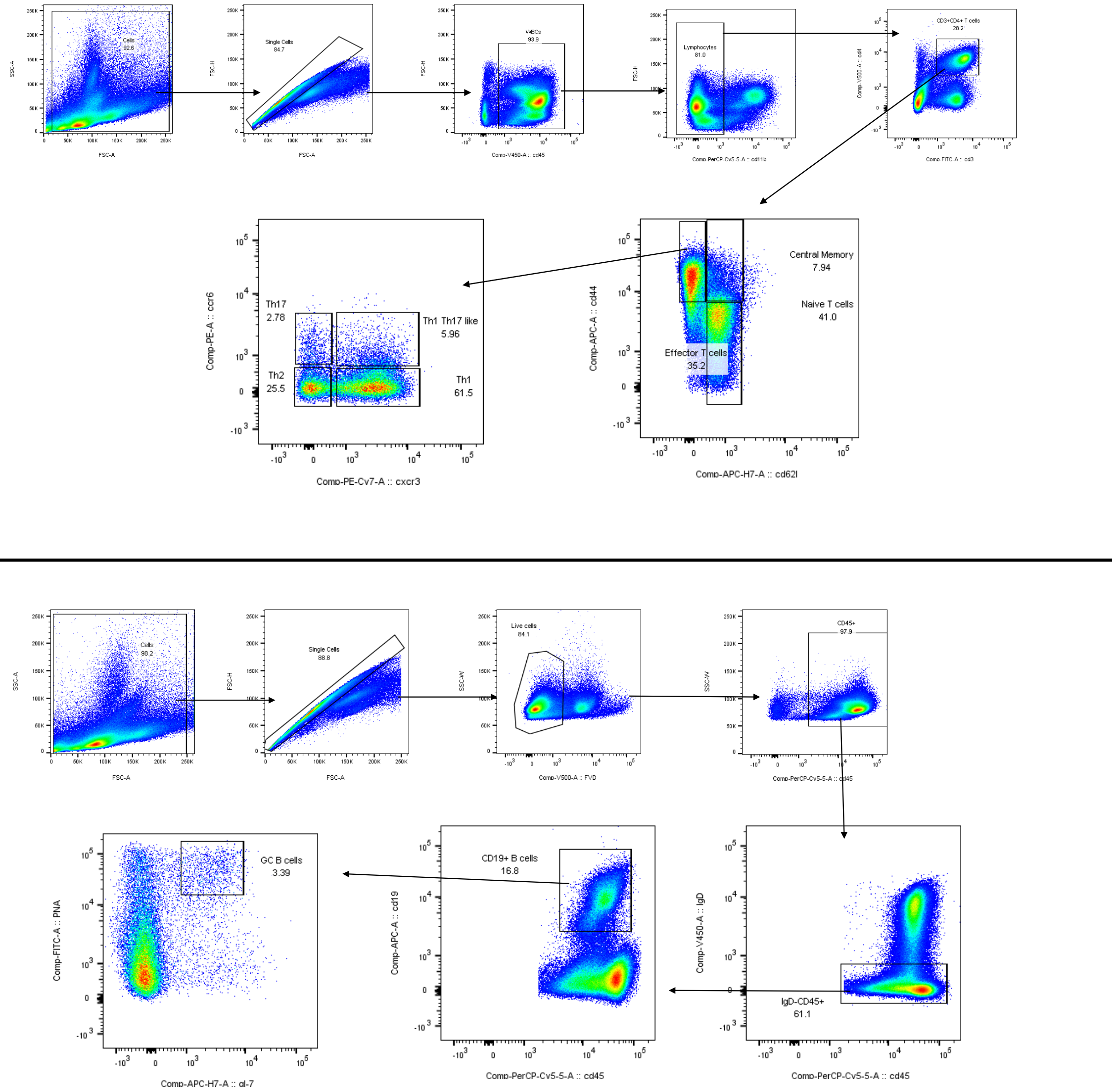

Supplementary Figure 5 Representative gating strategies. Flow cytometry panels used for gating T helper cell subsets are displayed in the top panel and panels for gating germinal center B cells are displayed in the bottom panel.

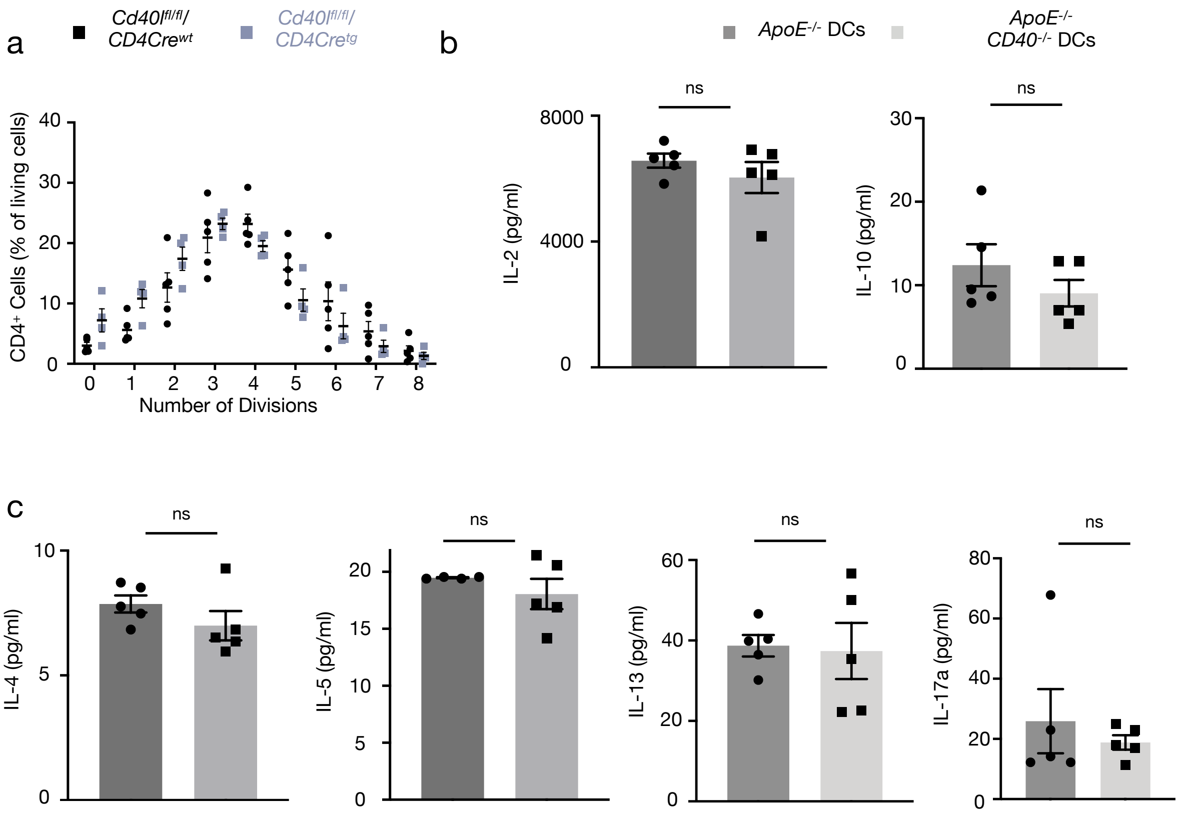

Supplementary Figure 6 T cell CD40L deficiency does not affect antigen-independent specific proliferation, but antigen-specific activation selectively inhibits a Th1 response. **a** Non-antigen specific T cell proliferation assay utilizing anti-CD3/CD28 beads to activate CFSE-stained $CD4^+$ T cells analyzed using CFSE dilution in living cells [n: WT=5, TG=4]. **b** Following antigen-specific T cell activation using OVA-primed CD40 wild type and CD40-deficient dendritic cells, Treg-associated cytokines including interleukin-2 (IL-2) and IL-10 were measured in the supernatant [n=5]. **c** Additional effector T cell cytokines including IL-4, IL-5, IL-13, and IL-17a were also measured in the supernatant of the DC-T cell co-culture [n=4-5]. Data are represented as mean ± s.e.m and n refers to biologically independent animals. Data was analyzed by a two-tailed unpaired Student's *t* test (**a-c**). Source data are provided as a Source Data file.

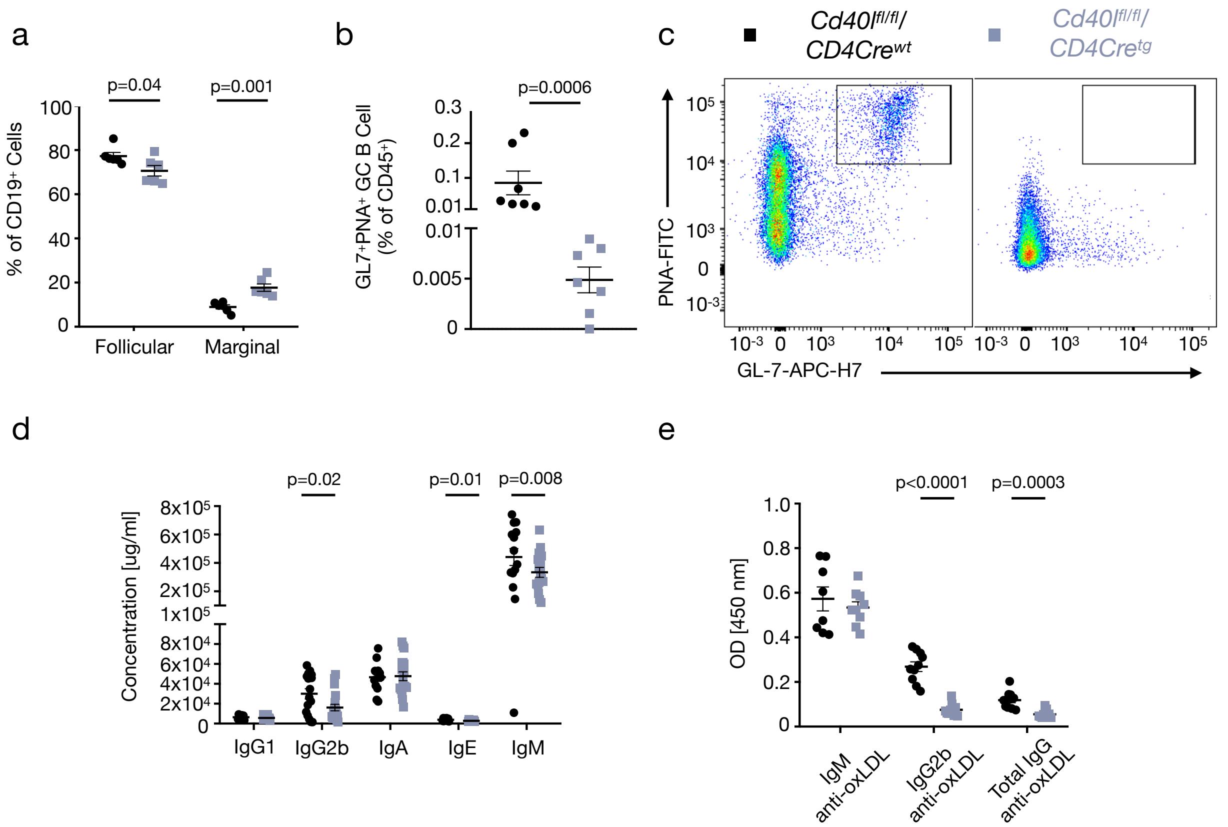

Supplementary Figure 7 T cell CD40L deficiency interferes with B cell development and function. **a-b,** Flow cytometric analysis of splenic B cell maturation stages of *Cd40l*$^{fl/fl}$*/Cd4Cre* mice including (**a**) follicular B cells and marginal zone B cells [n=6] and (**b**) germinal center (GC) B cells [n=7]. (**c**) Representative gating strategy for germinal centers in the spleen. **d-e**, Plasma concentrations of (**d**) general immunoglobulin titers including IgG1, IgG2b, IgA, IgE, and IgM [n: WT=16, TG=19] as well as (**e**) specific antibody titers directed against oxidized low-density lipoprotein (oxLDL) [n: WT=10, TG=9]. Data are represented as mean ± s.e.m and n refers to biologically independent animals. Data was analyzed by either a two-tailed unpaired Student's *t* test (**a, c-d**) or Mann Whitney test (**b-d**). Source data are provided as a Source Data file.

Supplementary Tables

Supplementary Table 1 General parameters of knockout models.

	$Cd40l^{fl/fl}$/ $Cd4Cre^{wt}$	$Cd40l^{fl/fl}$/ $CD4Cre^{tg}$	$Cd40l^{fl/fl}$/ $Pf4Cre^{wt}$	$Cd40l^{fl/fl}$/ $Pf4Cre^{tg}$	$Cd40^{fl/fl}$/ $Cd11cCre^{wt}$	$Cd40^{fl/fl}$/ $Cd11cCre^{tg}$
Body Weight (g)	33.42 ± 0.47	32.15 ± 0.52	33.73 ± 0.47	33.65 ± 0.52	30.00 ± 0.36	30.13 ± 0.48
Lymphocytes (%)	73.09 ± 1.18	73.88 ± 1.88	63.68 ± 2.27	66.71 ± 2.25	61.08 ± 3.51	61.58 ± 3.61
Monocytes (%)	4.03 ± 0.21	4.07 ± 0.20	4.49 ± 0.19	4.36 ± 0.23	5.27 ± 0.31	5.46 ± 0.49
Granulocytes (%)	22.87 ± 1.15	24.59 ± 2.24	31.84 ± 2.18	29.50 ± 2.15	33.66 ± 3.26	35.14 ± 3.37
Platelets (10^3/ul)	1275 ± 82	1218 ± 38	1323 ± 32	1280 ± 32	1228 ± 71	1265 ± 60
Erthyrocytes (10^6/ul)	8.68 ± 0.13	8.59 ± 0.11	8.33 ± 0.15	8.41 ± 0.24	8.65 ± 0.17	8.32 ± 0.17
Leukocytes (10^3/ul)	2.79 ± 0.17	3.57 ± 0.22	3.55 ± 0.18	3.64 ± 0.25	4.41 ± 0.39	3.78 ± 0.27
Lymphocytes (10^3/ul)	2.11 ± 0.20	2.76 ± 0.20	2.30 ± 0.13	2.35 ± 0.17	2.60 ± 0.25	2.16 ± 0.13
Monocytes (10^3/ul)	0.06 ± 0.01	0.09 ± 0.01	0.13 ± 0.01	0.11 ± 0.01	0.18 ± 0.02	0.16 ± 0.02
Granulocytes (10^3/ul)	0.77 ± 0.07	1.08 ± 0.12	1.39 ± 0.19	1.18 ± 0.15	1.63 ± 0.26	1.55 ± 0.21
Plasma Cholesterol [mmol]	2.60 ± 0.47	2.56 ± 0.42	3.97 ± 0.19	3.55 ± 0.20	3.59 ± 0.19	3.49 ± 0.26

Table legend. 11 parameters were compared between each knockout model and its respective wild type littermates to confirm no negative morphological consequences resulted from the individual knockouts. Data are represented as mean ± s.e.m and n refers to biologically independent animals. Source data are provided as a Source Data file.

Supplementary Table 2 Clinical characteristics of patients included in the Carotid Plaque Imaging Project (CPIP) cohort.

Baseline	**CPIP**<br>N=185
Sex, male/female (% female)	124/61 (33)
Age (IQR)	70 (IQR 64-75)
Smoking, currently or in the past (%)	81
Symptoms (%)	52
Diabetes (%)	30
Hypertension (%)	76
Use of statins (%)	88
Fasting lipoproteins, mmol/L (IQR)	
Total cholesterol	4.3 (3.5-5.1)
Triglycerides	1.3 (0.95-1.8)
LDL cholesterol	2.4 (1.9-3.2)
HDL cholesterol	1.1 (0.9-1.3)
HbA1c, mmol/mol (IQR)	43.7 (38.0-51.0)
CRP, mg/L (IQR)	3.0 (0.76-6.0)
Plaque-CD40, A.U./gram wet weight plaque (IQR)	32258812.7 (24004507.8-40551081.3)
Plaque-CD40L A.U./gram wet weight plaque (IQR)	177236.6 (132047.1-260715.2)
Blood-CD40 A.U./mL (IQR)	657.1 (539.3-894.5)
Blood-CD40L A.U./mL (IQR)	319.6 (127.6-568.1)

Table legend. IQR indicates Interquartile range, Hypertension indicates Systolic blood pressure >140 mm Hg or under anti-hypertensive treatment, LDL indicates Low density lipoprotein, HDL indicates High density lipoprotein, HbA1c indicates Hemoglobin A1c, CRP indicates C-reactive protein, A.U. indicates Arbitrary unit.

Supplementary Table 3 A complete list of all the flow cytometry antibodies used in this study.

Antibody / with fluorophore	Company	Clone	Dilution
Fc-block (anti-CD16/32)	eBiosciences	93	1:100
CD45 / APC-H7	eBiosciences	30-F11	1:400
CD11b /PerCP	eBiosciences	M1/70	1:100
Ly6g / PE	BD Biosciences	1A8	1:2000
CD115 / APC	Biolegend	AFS98	1:500
Ly6C / BV510	Biolegend	HK1.4	1:500
CD3e / FITC	eBiosciences	145-2C11	1:200
CD19 / eFluor450	eBiosciences	1D3	1:100
CD11c / Pe-Cy7	eBiosciences	N418	1:1000
MCHCII / APC-H7	eBiosciences	M5/114.15.2	1:800
CD3 / PerCP	eBiosciences	145-2C11	1:50
CD40 / V500	BD Biosciences	RM4-5	1:100
CD8a / eFluor450	eBiosciences	53-6.7	1:200
CD44 / APC	eBiosciences	IM7	1:1000
CD62L / Pe-Cy7	eBiosciences	Mel-14	1:800
CD25 / FitC	eBiosciences	PC61.5	1:200
FoxP3 / PE	eBiosciences	FJK-16S	1:40
GL-7 / Biotin	eBiosciences	GL-7	1:200
CD138	Biolegend	281-2	1:200
CD19 / APC	eBiosciences	1D3	1:200
B220 /Pe-Cy7	eBiosciences	RA3-6B2	1:200
PNA / FitC	Vector Labs		1:200
CD45 / PerCP	eBiosciences	30-F11	1:500
IgD / V450	eBiosciences	11-26	1:200
CD40 / PE	Biolegend	2/23	1:100
CD154 / PE	ThermoFisher	MR1	1:100
CD4 / APC-H7	BD Biosciences	GK1.5	1:100
CD8a / Pe-Cy7	eBiosciences	53-6.7	1:800

CD19 / PerCP	eBiosciences	1D3	1:100
CD11b / BV510	Biolegend	M1/70	1:500
CD45 / V450	eBiosciences	30-F11	1:400
CXCR3 / Pe-Cy7	Biolegend	CXCR3-173	1:100
CCR6 / PE	Biolegend	29-2L17	1:100
FoxP3 / eFluor450	eBiosciences	FJK-16S	1:40
Ki67 / FitC	eBiosciences	SolA15	1:100
CD62L / APC-H7	Biolegend	Mel-14	1:800
GL-7 / Biotin	eBiosciences	GL-7	1:200
Streptavin/ APC-eFlour780	eBiosciences	-	1:200

Supplementary Table 3 A complete list of all the primary immunohistochemistry antibodies used in this study.

Antibody	Company	Clone	Dilution
Mac3	BD Biosciences, San Jose, USA	M3/84	1:200
Alpha smooth muscle actin (a-SMA)	a-SMA, Sigma Aldrich	1A4	1:1000
CD3	BD Biosciences	145-2C11	1:100
CD4	BD Biosciences	RM4-5	1:100
Foxp3	eBiosciences, San Diego, USA	FJK-16S	1:50
CD11c	eBiosciences	N418	1:100
CD40	Biolegend, San Diego, USA	3/23	1:100
CD40L	Biolegend	MR1	1:100

Supplementary Table 5 A complete list of all the real time PCR primers used in this study.

Primers	Company	Number
IL-1ß	Thermo Fisher	Mm00434228_m1
IL-6	Thermo Fisher	Mm00446190_m1
IFN- γ	Thermo Fisher	Mm01168134_m1
TGF-ß	Thermo Fisher	Mm00441726_m1
IL-10	Thermo Fisher	Mm00439614_m1
TNF-α	Thermo Fisher	Mm00443258_m1
CD3	Thermo Fisher	Mm01179194_m1
CD69	Thermo Fisher	Mm01183378_m1
