# Supplementary material for: Cell-specific and divergent roles of the CD40L-CD40 axis in atherosclerotic vascular disease
Source: Nat Commun. 2021 Jun 18;12:3754. doi: 10.1038/s41467-021-23909-z (PMC8213756; doi:10.1038/s41467-021-23909-z)
Supplement: Supplementary file 3 — Reporting Summary [file 41467_2021_23909_MOESM3_ESM.pdf]

nature research

Corresponding author(s): Lutgens

Last updated by author(s): Mar 22, 2021

Reporting Summary

Nature Research wishes to improve the reproducibility of the work that we publish. This form provides structure for consistency and transparency in reporting. For further information on Nature Research policies, see our Editorial Policies and the Editorial Policy Checklist.

Statistics

For all statistical analyses, confirm that the following items are present in the figure legend, table legend, main text, or Methods section.

n/a	Confirmed	
☐	☒	The exact sample size (n) for each experimental group/condition, given as a discrete number and unit of measurement
☐	☒	A statement on whether measurements were taken from distinct samples or whether the same sample was measured repeatedly
☐	☒	The statistical test(s) used AND whether they are one- or two-sided<br>*Only common tests should be described solely by name; describe more complex techniques in the Methods section.*
☐	☒	A description of all covariates tested
☐	☒	A description of any assumptions or corrections, such as tests of normality and adjustment for multiple comparisons
☐	☒	A full description of the statistical parameters including central tendency (e.g. means) or other basic estimates (e.g. regression coefficient) AND variation (e.g. standard deviation) or associated estimates of uncertainty (e.g. confidence intervals)
☐	☒	For null hypothesis testing, the test statistic (e.g. F, t, r) with confidence intervals, effect sizes, degrees of freedom and P value noted<br>*Give P values as exact values whenever suitable.*
☒	☐	For Bayesian analysis, information on the choice of priors and Markov chain Monte Carlo settings
☒	☐	For hierarchical and complex designs, identification of the appropriate level for tests and full reporting of outcomes
☒	☐	Estimates of effect sizes (e.g. Cohen's d, Pearson's r), indicating how they were calculated

Our web collection on statistics for biologists contains articles on many of the points above.

Software and code

Policy information about availability of computer code

Data collection

FACS Canto II, FACSDiva software (Becton Dickinson, Heidelberg),
Leica DM6000, LAS 4.6 and LAS X, (Leica Microsystems, Wetzler, Germany),
MagPix, xPONENT 4.2.1324.0 (Luminex),
7900T Fast Real Time PCR system (Applied Biosystems, Thermo Fisher),
Microplate reader (Tecan)

Data analysis	GraphPad Prism 6, 7, version 7.0a, or version 8 (GraphPad Software Inc.),<br>FlowJo software v10 (FlowJo, Asheville, NC),<br>ImageJ (https://imagej.nih.gov/ij/index.html),<br>LAS 4.6 and LAS X, (Leica Microsystems, Wetzler, Germany),<br>Magellan v7.2 (Tecan),<br>DESeq2 1.20.0 (https://bioconductor.org/packages/release/bioc/html/DESeq2.html),<br>EGSEA 1.6.1 (https://bioconductor.statistik.tu-dortmund.de/packages/3.6/bioc/html/EGSEA.html),<br>STAR (2.5.2),<br>SAMtools (1.3.1),<br>HOMER (4.10),<br>Bioconductor (3.7)

For manuscripts utilizing custom algorithms or software that are central to the research but not yet described in published literature, software must be made available to editors and reviewers. We strongly encourage code deposition in a community repository (e.g. GitHub). See the Nature Research guidelines for submitting code & software for further information.

Data

Policy information about availability of data

All manuscripts must include a data availability statement. This statement should provide the following information, where applicable:

- Accession codes, unique identifiers, or web links for publicly available datasets
- A list of figures that have associated raw data
- A description of any restrictions on data availability

The data supporting the findings of this study are available within the paper and its Supplementary Information files. Due to regulations of the Swedish law, patient related data, even when unidentified by code, is considered sensitive data as long as the subjects are alive, and is allowed to be shared as individual data points. RNA sequencing data are publicly available under accession code [GSE145L585]. Source data are provided with this paper. All other data are available from the corresponding authors upon request.

Field-specific reporting

Please select the one below that is the best fit for your research. If you are not sure, read the appropriate sections before making your selection.

[x] Life sciences [ ] Behavioural & social sciences [ ] Ecological, evolutionary & environmental sciences

For a reference copy of the document with all sections, see nature.com/documents/nr-reporting-summary-flat.pdf

Life sciences study design

All studies must disclose on these points even when the disclosure is negative.

Sample size	Sample size calculations were not performed prior to experiments. Sample sizes were based on similar studies in the field. For example, atherosclerosis studies were performed with at least 10 biologically indepdent samples. For plaque phenotyping and immunohistochemistry, biologically independent samples were used with sizes at least 5 animals. All other experiments were performed with at least 3 distinct samples per group (i.e. 3 fully independent experiments).
Data exclusions	Data were excluded in two conditions. First, data were excluded when a sample failed due to technical issues. For example, in atherosclerosis studies, when plaque sections were deemed not useable due to improperly cut sections (ie. section folded in on itself). Second, data were excluded as outliers based on the mean of each experimental group. Individual data points were excluded if they were above or below two standard deviations of their group's mean. Exclusion criteria were established before data was collected. All exclusions are listed in the Source Data file.
Replication	All data produced was based on biologically independent samples. No technical replicates were used for statistical measurements. Mouse experiments, including organ harvesting for flow cytometry and immunohistochemistry staining, were repeated at least 2 times per cell-type knockout on different days. Data was compiled for analysis and found to be reproducible between experimental days.
Randomization	Mice were assigned to groups based on their genotype and were both age- and litter-matched.
Blinding	Experimentors were blinded in the atherosclerosis studies. Mice were assigned experimental numbers, which after analysis of plasma, immune cells, and lesion size were traced back to original numbers in order to perform statistical analysis. Similar precautions were taken with in vitro studies.

Reporting for specific materials, systems and methods

We require information from authors about some types of materials, experimental systems and methods used in many studies. Here, indicate whether each material, system or method listed is relevant to your study. If you are not sure if a list item applies to your research, read the appropriate section before selecting a response.

Materials & experimental systems

n/a	Involved in the study	
	x	Antibodies
x		Eukaryotic cell lines
x		Palaeontology and archaeology
	x	Animals and other organisms
	x	Human research participants
x		Clinical data
x		Dual use research of concern

Methods

n/a	Involved in the study	
x		ChIP-seq
	x	Flow cytometry
x		MRI-based neuroimaging

Antibodies

Antibodies used	Antibodies, including clone number and dilution, for both flow cytometry and immunohistochemistry are listed in Supplemental Tables S3 and S4, respectively.
Validation	All antibodies were validated by their manufacturer for the application (ie. flow cytometry, immunohistochemistry) prior to use in our experiments. For immunophenotyping of immune cells, commonly used markers for individual populations were chosen (ie. CD3 for T cells and CD19 for B cells).

Animals and other organisms

Policy information about studies involving animals; ARRIVE guidelines recommended for reporting animal research

Laboratory animals	Male mice were used throughout the study. Atherosclerosis studies were performed on Apoe-/- transgenic mice (stock No. 002052, Jackson Laboratory, Bar Harbor, ME, USA) using CD40fl/fl and CD40Lfl/fl mice with cell-specific knockouts throughout the use of CD11cCre (stock No: 008068, Jackson Laboratory, Bar Harbor, Maine, USA)59, Cd4Cre (stock No: 017336, Jackson Laboratory, Bar Harbor, Maine, USA)60 or Pf4Cre (stock No: 008535, Jackson Laboratory, Bar Harbor, Maine, USA) strains. Additionally, a global CD40 deficient line was used for the co-culture with OT-II (stock No. 004194, Jackson Laboratory, Bar Harbor, ME, USA) T cells. For flox mice, references 57 and 58 of the manuscript describe their generation and validation.
Wild animals	No wild animals were used in this study.
Field-collected samples	No field collected samples were used in this study.
Ethics oversight	Mice were bred and housed at the animal facility at Ludwig-Maximilians University of Munich following institutional guidelines. All animal experiments were approved by the local ethical committee for animal experimentation (TVA #55.2-1-54-2532-85-2014).

Note that full information on the approval of the study protocol must also be provided in the manuscript.

Human research participants

Policy information about studies involving human research participants

Population characteristics	Clinical characteristics of the patients are summarized in supplemental Table S2.
Recruitment	Blood samples and plaque tissue were obtained from 185 subjects of the Carotid Plaque Imaging Project (CPIP, Lund University) cohort, which consists of patients undergoing carotid endarterectomy at the Vascular Department of Skåne University Hospital (Malmö, Sweden). Samples were collected between 2005 and 2010. Indications to surgery were as described previously in reference #69 in the manuscript: patients with ipsilateral symptoms (amaurosis fugax, transient ischemia attack or stroke within 6 months prior to surgery) and a degree of stenosis >70% or without ipsilateral symptoms but with a degree of stenosis >80%. The degree of stenosis was assessed with doppler ultrasound based on flow velocities as previously validated in reference #70 of the manuscript.
Ethics oversight	Written informed consent was given by all patients and the study protocol was approved by the local Regional Ethical Committee (reference number 472/2005). The study fully conforms to the Declaration of Helsinki.

Note that full information on the approval of the study protocol must also be provided in the manuscript.

Flow Cytometry

Plots

Confirm that:

- [x] The axis labels state the marker and fluorochrome used (e.g. CD4-FITC).
- [x] The axis scales are clearly visible. Include numbers along axes only for bottom left plot of group (a 'group' is an analysis of identical markers).
- [x] All plots are contour plots with outliers or pseudocolor plots.
- [x] A numerical value for number of cells or percentage (with statistics) is provided.

Methodology

Sample preparation	Harvested spleens and lymph nodes were torn apart manually using forceps and subsequently filtered through a 70 µm stranger in order to prepare single cell suspensions. To lyse red blood cells, suspensions containing whole blood and spleens were first incubated with a red blood cell lysis buffer containing 150 mM ammonium chloride (Sigma Aldrich) and 10 mM sodium bicarbonate (Sigma Aldrich) at pH 7.4 for 2 minutes on ice. After incubation, cells were washed with PBS and plated for staining in microtiter plates (Costar 3799, Corning, Corning, USA). When necessary, cells were first stained with Fc-block (anti-CD16/32, eBiosciences, clone 93, 1:100) for 20 minutes on ice to prevent nonspecific binding. After washing, cells were stained with different combinations of antibodies depending on the experiment (see Supplemental Table S4 for clones).
Instrument	FACS Canto II (Becton Dickinson, Heidelberg)
Software	FACSDiva (Becton Dickinson, Heidelberg), FlowJo v10 (FlowJo, Asheville, NC)
Cell population abundance	Cells were only sorted in the Treg suppression assay where we isolated CD45+CD4+CD25+ expressing Tregs. FoxP3 was not chosen as a marker in this experiment as intracellular staining would leave the sorted cells unviable. Splenic Treg populations represented approximately 5-10% of all CD4+ cells.
Gating strategy	General immunophenotyping of immune cells began with analysis of forward and sideward scatter to eliminate debris. Doublets were then removed based on area and height discrimination in forward scatter. CD45 expression identified white blood cells in all isolated organs. Innate and adaptive immune cells were divided by CD11b expression. Innate cells were first divided into Ly6G+CD115- Neutrophils or Ly6G-CD115+ Monocytes. Furthermore, Monocytes were divided into classical or non-classical monocytes based on Ly6C expression. Adaptive immune cells were classified as either CD3-CD19+ B cells or CD3+CD19- T cells. T cells were then subclassified as either CD4+ or CD8+ T cells. CD4+ T cells could then be classified as CD44-CD62L+ Naive T cells, CD44+CD62L+ Central Memory T cells, CD44+CD62L- Effector Memory T cells, or FoxP3+CD25+ Tregs. Finally, Effector Memory T cells were then subdivided into helper T populations including CXCR3+CCR6- Th1, CXCR3-CCR6- Th2, and CXCR3-CCR6+ Th17 cells. Gating strategies are provided in Figure 3 and Supplemental Figure S5.

- [x] Tick this box to confirm that a figure exemplifying the gating strategy is provided in the Supplementary Information.
